# Cognitive Function and Subjective Well-Being in Japanese Community-Dwelling Older Adults: A Cross-Sectional Study

**DOI:** 10.3390/geriatrics10050120

**Published:** 2025-09-06

**Authors:** Baoxing Li, Tianshu Chu, Ziming Gong, Le Tian, Hiro Kishimoto

**Affiliations:** 1 Department of Behavior and Health Sciences, Graduate School of Human-Environment Studies, Kyushu University, Fukuoka 819-0395, Japan; li.baoxing.463@s.kyushu-u.ac.jp (B.L.); chutianshu_japan@yahoo.co.jp (T.C.); gong.ziming.835@s.kyushu-u.ac.jp (Z.G.); tian.le.627@s.kyushu-u.ac.jp (L.T.); 2 Faculty of Arts and Science, Kyushu University, Fukuoka 819-0395, Japan; 3 Center for Health Science and Counseling, Kyushu University, Fukuoka 819-0395, Japan

**Keywords:** subjective well-being, mild cognitive impairment, older adult

## Abstract

**Background**: The relationship between mild cognitive impairment (MCI) and subjective well-being remains poorly understood. We examined associations between cognitive function and well-being domains in community-dwelling older Japanese adults with and without MCI. **Subjects and Methods**: A cross-sectional analysis of 710 community-dwelling Japanese adults aged 65–75 years was carried out. Well-being was measured using the Philadelphia Geriatric Center Morale Scale (PGCMS score ≥ 13 indicates high well-being), comprising agitation, attitude toward aging, and lonely dissatisfaction subscales. MCI was defined as a Montreal Cognitive Assessment (MoCA) score of 18–25. Multivariable logistic regression examined potential associations between socio-demographic and health factors. **Results**: Among the participants (mean age 70.0 ± 2.5 years, 49% women), 423 (59.6%) had MCI. The MCI status was not associated with overall well-being (OR 1.06, 95% CI: 0.72–1.57, *p* = 0.77). However, within the MCI group, each 1-point increase in the MoCA score was associated with lower agitation (OR 1.21, 95% CI: 1.04–1.41) but higher lonely dissatisfaction (OR 0.83, 95% CI: 0.70–0.98, *p* = 0.02). No associations were observed in the non-MCI group. **Conclusions**: Cognitive function shows domain-specific rather than global associations with well-being in individuals with MCI.

## 1. Introduction

As Japan continues to grapple with its super-aging population, the focus on promoting well-being in later life increased [1]. Subjective well-being is recognized as a component of overall well-being [2]. The field of subjective well-being encompasses the scientific analysis of how individuals evaluate their lives—both in the moment and over longer periods, such as over the prior year. These evaluations include individuals’ emotional responses to life events, their mood states, and cognitive judgments that individuals make about their life satisfaction, fulfillment, and satisfaction in specific domains such as marriage and work [2,3]. In gerontological research, subjective well-being is widely used as an indicator of successful aging [4]. Higher levels of subjective well-being are associated with better physical health outcomes and greater longevity [3]. Compared to objective indicators such as income and education, subjective well-being provides a more direct and comprehensive measure of individuals’ life evaluations [3,5]. According to the 2024 Survey Report on Well-being and Quality of Life in Japan, older adults consider health the most important factor influencing their subjective well-being [6]. As Japan’s aging population continues to grow, the maintenance of physical and mental well-being has emerged as a crucial public health priority.

Cognitive function is known to be closely associated with health outcomes in older adults, including declines in activities of daily living (ADLs) [7] and an increased mortality risk [8]. Mild cognitive impairment (MCI) is generally defined as subjective and objective cognitive decline that does not meet the diagnostic criteria for dementia, characterized by impairments in learning new information or recalling stored information [9,10]. The prevalence of MCI among individuals aged ≥65 years is estimated to be 3–22%, depending on population demographics [10]. Regional studies conducted between 2022 and 2023 in Hisayama Town (Fukuoka Prefecture), Nakajima Town (Ishikawa Prefecture), Nakayama Town (Ehime Prefecture), and Ama Town (Shimane Prefecture) reported a 15.5% prevalence of MCI among the study participants aged ≥65 years [11]. Factors associated with a greater likelihood of returning to normal cognition include the absence of depression and the use of anticholinergic medications [10].

It has been speculated that higher cognitive function in older adults may be associated with higher levels of subjective well-being. However, findings from some cross-sectional studies are not consistent with this speculation, potentially due to differences in the methods used to measure subjective well-being and/or variations in cultural values [12].

Given the abstract nature of subjective well-being as a construct, criticism has been directed at single-item measures of subjective well-being, suggesting that such measures may fail to fully capture its complexity [13]. Multidimensional scales are thought to provide a more comprehensive evaluation of subjective well-being. For example, the Philadelphia Geriatric Center Morale Scale (PGCMS) is a useful scale for measuring the subjective well-being of older adults from multiple perspectives [12]. Existing literature has overlooked multidimensional assessments of well-being among community-dwelling older adults. This study addresses this gap by applying a measure of subjective well-being in this population.

To date, few studies have examined the relationship between MCI and subjective well-being in community-dwelling older adults by using multidimensional assessment tools. It has been demonstrated that higher levels of subjective well-being are associated with a greater survival rate, regardless of disease status [14]. If cognitive function significantly influences subjective well-being, it is essential to prioritize the maintenance of cognitive abilities not merely for extending lifespan but also to preserve older adults’ independence and enhance their quality of life.

This study aims to examine the association between mild cognitive impairment (MCI) and subjective well-being among older community-dwelling adults. According to Freund’s view of successful aging as the optimal use of internal resources to manage aging-related losses, cognitive function plays a central role in preserving well-being in later life [15]. Informed by this perspective, we hypothesized that older adults without MCI would exhibit higher levels of subjective well-being, experience less loneliness and anxiety, and report fewer difficulties in daily activities compared to those with MCI. We also hypothesized that higher cognitive function would be positively associated with better subjective well-being.

## 2. Materials and Methods

### 2.1. Study Design and Participants

This was a population-based cross-sectional epidemiological study conducted in 2017 in the city of Itoshima, Japan, examining modifiable lifestyle and social factors. The inclusion criteria were as follows: residents of Itoshima City; respondents to the 2016 Itoshima Prefectural Needs Survey; aged 65–75 years who were not certified as requiring nursing care by the Japanese National Long-term Care Insurance System in 2017.

We used a software program to randomly select 5000 subjects from among the approx. 10,000 older adults in Itoshima, stratified by residential area, sex, and age. Study information sheets and a questionnaire were mailed to these individuals, inviting them to community centers for further assessments from September to December 2017, during which cognitive function was measured using both the Montreal Cognitive Assessment (MoCA) and the Mini-Mental State Examination (MMSE). Participants were instructed to complete the questionnaire by themselves whenever possible. If they experienced difficulty, they were advised to seek assistance from research staff during the on-site assessment.

Of the 5000 individuals contacted, 1589 submitted the questionnaires (31.8% response rate), and 949 participated in further assessments. As the present study focused on older adults aged 65–75 years, we excluded 146 individuals who fell outside this age range. We then excluded 16 candidate participants whose MoCA data were incomplete (due to missing responses making the total score calculation impossible) and another 18 candidates with MoCA scores ≤ 17 points (based on a prior study using <18 points as the cutoff for cognitive impairment worse than MCI, possibly indicating dementia) [16].

**Figure 1:**
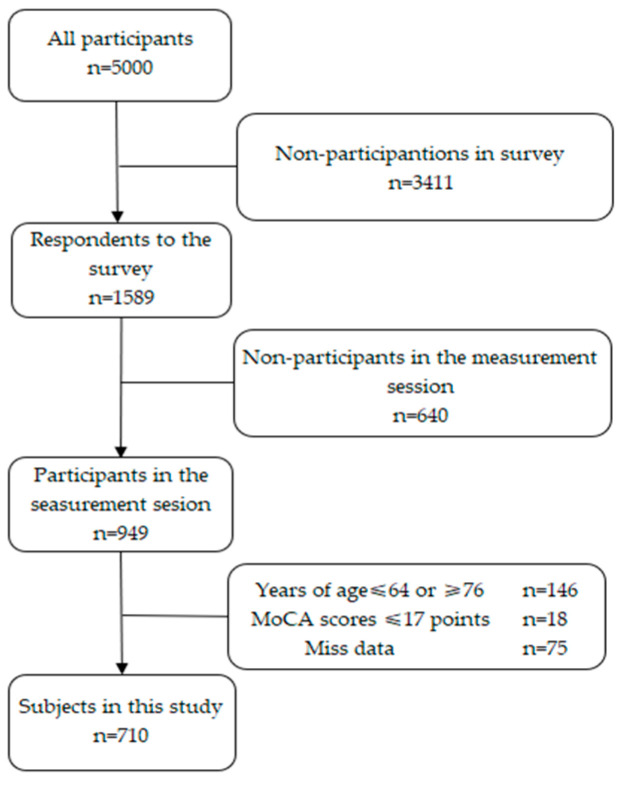
Participant flow chart in the analysis.

Additional exclusions due to the absence of data listed were as follows: subjective well-being data (*n* = 29), the number of education years (*n* = 3), residential duration information (*n* = 1), subjective economic status (*n* = 4), alcohol consumption information (*n* = 1), tobacco smoking history (*n* = 1), number of natural teeth (*n* = 1), meat and fish intake frequency (*n* = 3), and Kessler 6 (K6) Scale data (*n* = 16). The final analyses thus included 710 participants (Figure 1).

Regarding the assessment of MCI, it had been suggested that individuals with an MMSE score ≤ 12 points may not be able to provide accurate or reliable responses to self-reported questions [17]. All 710 participants in the present study had MMSE scores > 12 points. The study was approved by the Institutional Review Board of Kyushu University (approval no. 201708), and written informed consent was obtained from all participants [18].

### 2.2. Measurements

#### 2.2.1. Measurement of Subjective Well-Being

The Philadelphia Geriatric Center Morale Scale (PGCMS) was developed to measure a generalized feeling of well-being based on various indicators such as freedom from distressing symptoms, satisfaction with self, a sense of harmony between self and environment, and the ability to strive appropriately while accepting the inevitable. The PGCMS was translated into Japanese in 1979, and its psychometric properties, including reliability and validity, have since been demonstrated in Japanese populations. It is widely used in Japan and has been recommended by the United Kingdom’s Royal College of Physicians and the British Geriatric Society for measuring subjective well-being [4,12,19,20,21].

We used the 17-item PGCMS to assess subjective well-being. Scores of 13–17 points indicate high levels of well-being, 10–12 points indicate a midrange, and scores of ≤9 points indicate a low level of subjective well-being [20]. The PGCMS consists of three dimensions: agitation, attitude toward one’s own aging, and lonely dissatisfaction [12,19,20]. The agitation dimension has six items that assess anxiety experienced by older adults; the ‘attitude toward one’s own aging’ dimension comprises five items capturing an individual’s perceptions and evaluations of changes occurring in their lives; and the ‘lonely dissatisfaction’ dimension has six items reflecting the acceptance of or dissatisfaction with the individual’s current level of social interaction. In this study, we analyzed not only the total PGCMS score but also associations between cognitive function and each of the scale’s three dimensions, i.e., subscales.

#### 2.2.2. MCI Measured by the MoCA

The Montreal Cognitive Assessment (MoCA) is a brief screening tool with high sensitivity and specificity for detecting MCI among individuals who perform in the normal range on the MMSE [22]. The total possible score ranges from 0 to 30 points [16]. In this study, a MoCA score of 18–25 points was defined as indicating MCI, which is consistent with the definition of MCI as a stage of impairment that does not meet the diagnostic criteria for dementia [16]. The MoCA assesses multiple cognitive domains including memory, visuospatial abilities, executive functions, attention, language, and orientation, with higher scores indicating better cognitive performance in each domain [23].

#### 2.2.3. Other Survey Items

Self-reported data were collected via questionnaires for the following variables: gender, age, the length of time that the respondent had been living at his/her residence (residential duration), number of years of education, sleep quality, mental health, instrumental activities of daily living (IADLs), subjective financial status, subjective health status, tobacco smoking history, alcohol consumption, number of natural teeth, monthly frequency of meat/fish consumption, and monthly frequency of vegetable/fruit consumption. Residential duration was assessed by asking, “How many years have you lived at your current address?” and years of education were collected by asking, “How many years of formal education did you receive in your youth?”

Sleep quality was assessed using the Pittsburgh Sleep Quality Index (PSQI), which includes seven subscales: sleep quality, sleep latency, sleep duration, sleep efficiency, sleep disturbances, use of sleeping medication, and daytime dysfunction. The total possible scores on the PSQI range from 0 to 21 points, with ≥6 points indicating poor sleep quality [24]. The participants’ IADLs were evaluated using the 13-item Tokyo Metropolitan Institute of Gerontology Index of Competence; each ‘yes’ response was scored as 1 point and each ‘no’ response was scored as 0 points. The total possible score ranges from 0 to 13 points, with higher scores indicating better functioning [25]. The participants’ mental health was assessed using the K6 scale, which evaluates depressive mood and anxiety over the past 30 days, with five response options: ‘none,’ ‘a little,’ ‘sometimes,’ ‘mostly,’ and ‘always.’ The possible score ranges from 0 to 24 points, with higher scores indicating greater psychological distress. Participants with scores ≥ 6 points were defined as having significant depressive mood and anxiety [26,27].

The subjective financial status of the participants was assessed by asking, “How do you feel about your current financial situation?” with the response options ‘somewhat comfortable,’ ‘comfortable,’ ‘difficult,’ and ‘somewhat difficult.’ The ‘somewhat comfortable’ and ‘comfortable’ responses were categorized as financially comfortable. The subjective health status was assessed by asking “How do you consider your health?” with the options ‘very healthy,’ ‘moderately healthy,’ ‘not very healthy,’ and ‘unhealthy.’ The ‘very healthy’ and ‘moderately healthy’ responses were categorized as healthy. The participant’s tobacco smoking history was assessed by asking, “Do you smoke cigarettes?” with these options: ‘quit smoking,’ ‘never smoked,’ ‘smoke almost daily,’ and ‘smoke occasionally.’ The participants who gave the ‘quit smoking’ and ‘never smoked’ response were categorized as non-smokers. Alcohol consumption was assessed by asking, “Do you drink alcohol, and if so, how often?” The participants who gave the ‘rarely drink’ or ‘never drink’ response were categorized as non-drinkers. The numbers of natural teeth were categorized into 0, 1–9, 10–19, and ≥20, and then dichotomized into ≥20 versus <20. Monthly meat and fish consumption frequency was assessed with the options ‘twice daily or more,’ ‘once daily,’ ‘4–6 times weekly,’ and so on, and dichotomized into ‘once daily or more’ versus ‘less than once daily.’ Monthly vegetable and fruit consumption frequency was assessed in the same way and dichotomized into ‘twice daily or more’ versus ‘less than twice daily.’

### 2.3. Statistical Analyses

The associations between cognitive function scores and subjective well-being as measured by the PGCMS including its three subscales (agitation, attitude toward one’s own aging, lonely dissatisfaction) were examined with the use of scatter plots, and Pearson’s correlation coefficients were calculated. We divided the participants into two groups based on their MoCA scores: the MCI group (score 18–25 points, *n* = 423) and the non-MCI group (score ≥ 26 points, *n* = 287). Group characteristics were compared by the chi-square test or unpaired *t*-tests. We performed a logistic regression to calculate odds ratios (ORs) with 95% confidence intervals (CIs) to analyze the proportion and odds of higher subjective well-being in the non-MCI group (reference: MCI group). The covariates adjusted in the multivariate analysis included age, gender, years of education, residential duration, subjective financial status, subjective health status, mental health, IADLs, sleep quality, tobacco smoking history, alcohol consumption, number of natural teeth, frequency of meat and fish consumption, and frequency of vegetable and fruit consumption. To examine the potential linear dose–response relationships between the participants’ MCI status and their subjective well-being (including the PGCMS subscales), we calculated the proportion and probability of older adults with high subjective well-being for each 1-point increase in the PGCMS cognitive function score. The statistical significance level was set at α = 0.05. All analyses were performed using SAS ver. 9.4 (SAS Institute, Cary, NC, USA).

## 3. Results

### 3.1. Characteristics of the MCI and Non-MCI Groups

The participants’ overall mean age was 70 years, and 49% were women. The mean cognitive function score was 24.6 points. There were 287 participants with normal cognitive function. The mean subjective well-being score was 12.7 points, with the participants categorized as having low (*n* = 114), moderate (*n* = 167), and high (*n* = 429) subjective well-being.

As shown in Table 1, compared to the MCI group, the non-MCI group was younger and had shorter residential durations, more years of education, a higher proportion of women, a greater number of natural teeth, and higher monthly consumption frequencies of meat, fish, vegetables, and fruits (all *p* < 0.05).

No significant differences were observed between the MCI and non-MCI groups in total PGCMS scores or the agitation score. The non-MCI group tended to have lower scores on the lonely dissatisfaction subscale (*p* = 0.07), but it demonstrated significantly higher scores for attitudes toward their own aging (*p* = 0.049). No other significant differences were observed between the MCI and non-MCI groups.

### 3.2. Odds Ratios of Subjective Well-Being and the PGCMS Subscales by MCI Status

Table 2 presents the prevalence rates and adjusted ORs (95% CIs) of subjective well-being and its subscales between the MCI and non-MCI groups, based on logistic regression. The prevalence of high subjective well-being (defined as a PGCMS score ≥ 13 points) was 59.1% (*n* = 250) in the MCI group and 62.4% (*n* = 179) in the non-MCI group.

After the adjustment for age, gender, years of education, residential duration, subjective financial status, subjective health status, mental health, IADLs, sleep quality, tobacco smoking history, alcohol consumption, number of natural teeth, and consumption frequencies of meat, fish, vegetables, and fruits, the OR (95% CI: *p*-value) for high subjective well-being in the non-MCI group (reference: MCI group) was 1.06 (95% CI: 0.72–1.57, *p* = 0.77), indicating no significant association. Similarly, the OR per 1-point increase in the PGCMS cognitive function score was 0.99 (95% CI: 0.92–1.07, *p* = 0.76).

No significant associations were observed between the MCI status and any of the PGCMS subscales of subjective well-being.

### 3.3. Multivariable-Adjusted ORs of Subjective Well-Being per 1-Point Increase on the Cognitive Function by MCI Status

Table 3 shows the ORs (95% CIs) of subjective well-being on the PGCMS and its subscales per 1-point increase in the score on the cognitive function based on logistic regression stratified by the participants’ MCI status. In the MCI group, each 1-point increase in cognitive function was significantly associated with higher odds of lower agitation (OR 1.21, 95% CI: 1.04–1.41, *p* = 0.02) but was significantly associated with lower odds of reduced lonely dissatisfaction (OR 0.83, 95% CI: 0.70–0.98, *p* = 0.03). No other significant associations were observed. In contrast, the non-MCI group showed no significant association between cognitive function and subjective well-being or any of the PGCMS subscales.

## 4. Discussion

Our analyses identified no consistent correlation or linear relationship between cognitive function and subjective well-being (including its subscales) in the study participants, all of whom were older community-dwelling Japanese adults. The inconsistency between the result and our initial hypothesis may be partially explained by differences in the conceptualization and measurement of subjective well-being. Wilson et al. conducted a longitudinal study tracking older adults with normal baseline cognition, measuring their cognitive function across five domains (episodic memory, semantic memory, working memory, perceptual speed, and visuospatial ability) and well-being using six dimensions from Ryff’s Psychological Well-Being Scale (autonomy, environmental mastery, personal growth, positive relations, purpose in life, and self-acceptance) [28]. They reported that faster cognitive decline was correlated with deterioration in five of the six well-being dimensions; self-acceptance was the exception. The PGCMS, which we used to assess subjective well-being in the present study, reflects satisfaction with oneself and the ability to strive appropriately while still accepting the inevitable [12]. Furthermore, the lack of statistically significant differences in our findings may be attributed to insufficient statistical power due to the relatively small sample size, as indicated by our post hoc power analysis.

We used the MoCA scores to classify the participants into MCI and non-MCI groups. The progression from normal cognition to MCI has been reported to manifest primarily as reduced performance on cognitive tests, without reaching the threshold of functional impairment [29]. Although the average MoCA score falls within the MCI range, the absence of functional impairments and the independent living status of our participants suggest that they had likely not yet experienced clinically significant cognitive decline.

We could not conclude that the present non-MCI group had significantly higher proportions of participants with greater subjective well-being, lower agitation, more positive attitudes toward their own aging, or a lower level of lonely dissatisfaction compared to the MCI group. Cooper et al. reported cross-sectional findings from 14,769 adults aged ≥65 years in low- and middle-income countries, investigating relationships between cognitive function and happiness, and how social networks mediate this association [30]. In that study, cognitive function was assessed using the Community Screening Interview for Dementia, and happiness was measured by a single question (“How happy do you feel?”) with four response options: ‘very happy,’ ‘moderately happy,’ ‘not very happy,’ and ‘not happy at all.’ An ordinal logistic regression model was adjusted for the evaluation of demographic characteristics, social network type, cognitive function scores, presence of depressive symptoms, physical disabilities, and interaction terms between the social network type and cognitive function. Their results indicated that the social network type mediated the relationship between cognitive function and happiness, whereas the cognitive scores themselves showed no significant direct association with happiness [30]. Our findings support Cooper et al.’s results. After the adjustment for similar variables including demographics, subjective economic status, subjective health status, mental health, IADLs, sleep quality, tobacco smoking history, alcohol consumption, number of natural teeth, and dietary habits, there were no significant differences between the MCI and non-MCI groups in ORs for subjective well-being or its subscales on the PGCMS.

In the MCI group, each 1-point increase in the cognitive function score was associated with significantly higher odds of having higher agitation (OR 1.21, 95% CI: 1.04–1.41, *p* = 0.02). The agitation subscale of the PGCMS significantly reflects anxiety experienced by older adults, including worries and irritability. Yochim et al. evaluated the association between anxiety symptoms and memory and executive function in 120 community-dwelling older adults [31]; their participants completed the Geriatric Anxiety Scale, the California Verbal Learning Test (CVLT), and the Delis–Kaplan Executive Function System (D-KEFS). A multiple regression analysis showed that anxiety predicted poorer performance on both CVLT-measured memory and D-KEFS-measured executive function. In addition, Frankenberg et al. demonstrated that higher word counts on verbal fluency tests correlated with more detailed life-experience narratives in autobiographical interviews, suggesting a tendency for spontaneous, rapid speech in daily life [32]. This implies that differences in the quality and quantity of social interactions, and thus linguistic ability, may relate to older adults’ sense of purpose and well-being [33].

In our present study’s MCI group, each 1-point increase in the cognitive function score was associated with higher odds of experiencing lonely dissatisfaction. This result contrasts with our initial hypothesis that higher cognitive function would correlate with lower lonely dissatisfaction. We had measured lonely dissatisfaction based on feelings of loneliness, harsh emotions, interactions with family and friends, and life satisfaction. Using data from an international social survey, Bellucci et al. conducted a cross-sectional study of 15,430 participants across 15 countries [34]. Their findings revealed that loneliness assessed through feelings of having no companions, social isolation, and being excluded is associated with strong motivations for prosocial behaviors such as helping and supporting others, suggesting that loneliness may promote healthy social bonding by increasing the desire for connections, ultimately reflecting more positive social behaviors. However, the lonely dissatisfaction subscale of the PGCMS may not fully capture the multidimensional nature of loneliness as conceptualized in broader social and psychological literature. Given this limitation, our study can only cautiously interpret the relationship between cognitive function and loneliness, and further research is needed to explore the complex mechanisms underlying this association.

This study has several limitations. As a cross-sectional design, it cannot establish causal relationships between cognitive function and subjective well-being; however, it was necessary to examine their association given the lack of baseline data. In addition, our participants’ cognitive function was assessed only via global MoCA scores, leaving unclear how specific cognitive domains such as language, executive function, and memory relate to subjective well-being. Future research could also examine potential mediating factors between cognitive function and well-being, such as physical fitness, physical activity, and social engagement. Finally, the reliance on self-reported measures of subjective well-being may introduce recall and/or response bias. Future studies may benefit from incorporating qualitative methods to gain deeper insight into individual perceptions and experiences. From a clinical perspective, incorporating both cognitive assessments and measures of emotional well-being in routine screenings may enhance early identification of older adults at risk of declining subjective well-being, enabling timely support through psychosocial interventions.

## 5. Conclusions

We found no evidence that higher cognitive function is associated with greater subjective well-being in community-dwelling older adults. Cognitive function showed differential associations with subjective well-being subscales: The participants with mild cognitive impairment and higher MoCA scores had lower agitation but reported greater lonely dissatisfaction.

## Figures and Tables

**Table 1:** Characteristics of the MCI and non-MCI groups.

	MCI *n* = 423	Non-MCI *n* = 287	*p*-Value
Age, yrs	70.1 ± 2.6	69.6 ± 2.3	0.01
Women (%)	191 (45.2)	159 (55.4)	0.01
Education, yrs	12.8 ± 2.4	13.5 ± 2.4	<0.01
Residential duration, yrs	30.1 ± 18.0	26.2 ± 15.4	<0.01
IADLs score, points	12.2 ± 1.1	12.3 ± 1.1	0.06
K6 score, points	2.4 ± 3.2	2.1 ± 2.3	0.07
PSQI score, points	4.0 ± 2.8	3.9 ± 2.6	0.72
Financially comfortable status	203 (48.0)	143 (49.8)	0.63
Positive subjective health status	47 (11.1)	31 (10.8)	0.90
Non-tobacco smokers	380 (89.8)	266 (92.7)	0.19
Non-alcohol consumers	191 (45.2)	146 (50.9)	0.13
≥20 natural teeth	210 (50.0)	165 (57.5)	0.04
Meat and fish consumption frequency, ≥1×/day	243 (57.5)	196 (68.3)	<0.01
Vegetable and fruit consumption frequency, ≥2×/day	198 (46.8)	180 (62.7)	<0.01
MoCA score ≥ 20 points	23.0 ± 1.8	27.1 ± 1.09	<0.01
Subjective well-being score, points	12.5 ± 3.7	12.9 ± 3.4	0.18
Agitation score, points	4.6 ± 1.6	4.6 ± 1.5	0.76
Attitude toward own aging score, points	3.3 ± 1.4	3.5 ± 1.4	0.049
Lonely dissatisfaction score, points	4.6 ± 1.4	4.8 ± 1.3	0.07

Data are mean ± standard deviation, or *n* (%). MCI group: MoCA scores of 18–25 points. IADLs: instrumental activities of daily living, K6: Kessler-6 Scale, MoCA: Montreal Cognitive Assessment, PSQI: The Pittsburgh Sleep Quality Index.

**Table 2:** Multivariable-adjusted odds ratios of subjective well-being and its subscales by cognitive function score.

	MCI *n* = 423	Non-MCI *n* = 287	Each 1-Point Increase in the Cognitive Function Score
High subjective well-being *			
* n* (%)	250 (59.1)	179 (62.4)	–
OR	1.00	1.06	0.99
95% CI	Ref.	0.72–1.57	0.92–1.07
*p*-value	–	0.77	0.76
Low agitation **			
*n* (%)	267 (63.1)	172 (59.9)	–
OR	1.00	0.75	1.01
95% CI	Ref.	0.50–1.15	0.94–1.10
*p*-value	–	0.20	0.74
Positive attitude toward one’s own aging **			
*n* (%)	217 (51.3)	172 (59.9)	–
OR	1.00	1.34	1.08
95% CI	Ref.	0.89–2.04	1.00–1.12
*p*-value	–	0.17	0.06
Low lonely dissatisfaction **			
*n* (%)	276 (65.3)	192 (66.9)	–
OR	1.00	1.05	0.96
95% CI	Ref.	0.67–1.63	0.88–1.04
*p*-value	–	0.84	0.29

* Adjusted for gender, age, residential duration, education years, sleep quality, mental health, IADLs, subjective economic status, subjective health status, tobacco smoking history, alcohol consumption, number of natural teeth, monthly frequency of meat and fish consumption, and monthly frequency of vegetable and fruit consumption. ** Adjusted for gender, age, residential duration, education years, sleep quality, mental health, IADLs, subjective economic status, subjective health status, tobacco smoking history, alcohol consumption, number of natural teeth, monthly frequency of meat and fish consumption, and monthly frequency of vegetable and fruit consumption. High subjective well-being: defined as a PGCMS score ≥ 13 points; Low agitation: agitation score ≥ 5 points; Low lonely dissatisfaction: lonely dissatisfaction score ≥ 5 points; MCI group: MoCA score 18–25 points; Positive attitude toward one’s own aging: attitude toward one’s own aging score ≥ 4 points.

**Table 3:** Multivariable-adjusted odds ratios for subjective well-being per 1-point cognitive score increase in MCI and non-MCI groups.

	OR	95% CI	*p*-Value
**MCI group:**			
High subjective well-being *	0.93	0.81–1.07	0.33
Low agitation **	1.21	1.04–1.41	0.02
Positive attitude toward own aging **	1.06	0.91–1.23	0.49
Low lonely dissatisfaction **	0.83	0.70–0.98	0.03
**Non-MCI group:**			
High subjective well-being *	0.94	0.72–1.24	0.67
Low agitation **	1.05	0.79–1.41	0.73
Positive attitude toward own aging **	1.24	0.94–1.64	0.13
Low lonely dissatisfaction **	0.92	0.67–1.26	0.60

* Adjusted for gender, age, residential duration, education years, sleep quality, mental health, instrumental ADLs, subjective economic status, subjective health status, tobacco smoking history, alcohol consumption, number of natural teeth, monthly frequency of meat and fish consumption, and monthly frequency of vegetable and fruit consumption. ** Adjusted for gender, age, residential duration, education years, sleep quality, mental health, instrumental ADLs, subjective economic status, subjective health status, tobacco smoking history, alcohol consumption, number of natural teeth, monthly frequency of meat and fish consumption, and monthly frequency of vegetable and fruit consumption. High subjective well-being: defined as a PGCMS score ≥ 13 points; Low agitation: agitation score ≥ 5 points; Low lonely dissatisfaction: lonely dissatisfaction score ≥ 5 points; MCI group: MoCA scores 18–25 points; Positive attitude toward one’s own aging: attitude toward one’s own aging score ≥ 4 points.

## Data Availability

No new data were created or analyzed in this study. Data sharing is not applicable to this article.

## Abbreviations

IADLs	instrumental activities of daily living
K6	Kessler Psychological Distress Scale
MCI	mild cognitive impairment
MMSE	Mini-Mental State Examination
MoCA	The Montreal Cognitive Assessment
PSQI	Pittsburgh Sleep Quality Index

## References

[1] Saito T. (2024). Contemporary aspects of multigenerational living arrangements in an aging society: Examining the well-being of older adults in Japan. J. Soc. Secur. Res..

[2] Diener E., Oishi S., Lucas R.E. (2003). Personality, culture, and subjective well-being: Emotional and cognitive evaluations of life. Annu. Rev. Psychol..

[3] Diener E., Chan M.Y. (2011). Happy people live longer: Subjective well-being contributes to health and longevity. Appl. Psychol. Health Well-Being.

[4] Nakagawa T. (2018). Stability and change in subjective well-being in old age. Jpn. J. Gerontol..

[5] Douma L., Steverink N., Hutter I., Meijering L. (2017). Exploring subjective well-being in older age by using participant-generated word clouds. Gerontologist.

[6] Cabinet Office (2024). Well-being and Quality of Life Survey Report. https://www5.cao.go.jp/keizai2/wellbeing/manzoku/pdf/summary24.pdf.

[7] Hotta R., Fujiwara H., Hashimoto K. (2010). Is cognitive function in elderly associated with activities in daily life. J-STAGE.

[8] Kokubo k Horiguchi M., Mori T. (2020). Life prognosis of persons with dementia: Observation of persons registered for long-term care insurance in a community in Japan. Nihon Koshu Eisei Zasshi.

[9] Kenkyusha Co., Ltd. (2008). English-Japanese Dictionary of Medical Science.

[10] Sanford A.M. (2017). Mild cognitive impairment. Clin. Geriatr. Med..

[11] Ninomiya T. (2023). Ministry of Health, Labour and Welfare Sciences Research Grant, Administrative Policy Research Area, Ministry of Health, Labour and Welfare Sciences Special Research: Research on future estimates of the elderly dementia population in Japan. https://www.eph.med.kyushu-u.ac.jp/jpsc/uploads/resmaterials/0000000111.pdf?1715072186.

[12] Liang J., Bollen K.A. (1983). The structure of the Philadelphia Geriatric Center Morale scale: A reinterpretation. J. Gerotol..

[13] Tan J.H., Abdin E., Shahwan S., Zhang Y., Sambasivam R., Vaingankar J.A., Mahendran R., Chua H.C., Chong S.A., Subramaniam M. (2019). Happiness and cognitive impairment among older adults: Investigating the mediational roles of disability, depression, social contact frequency, and loneliness. Int. J. Environ. Res. Public Health.

[14] Watanabe H., Megumi A., Yasumura A. (2021). Relation between subjective well-being and cognitive function in older adults. Kumamoto Univ. Stud. Soc. Cult. Sci..

[15] Freund A.M. (2008). Successful aging as management of resources: The role of selection, optimization, and compensation. Res. Hum. Dev..

[16] Hoops S., Nazem S., Siderowf A.D., Duda J.E., Xie S.X., Stern M.B., Weintraub D. (2009). Validity of the MoCA and MMSE in the detection of MCI and dementia in Parkinson disease. Neurology.

[17] Leggett A., Zarit S.H., Hoang C.N., Nguyen H.T. (2013). Correlates of cognitive impairment in older Vietnamese. Aging Ment. Health.

[18] Yokote T., Yatsugi H., Chu T., Liu X., Wang L., Kishimoto H. (2024). Association of the combination of moderate-to-vigorous physical activity and sleep quality with physical frailty. Geriatrics.

[19] Niklasson J., Conradsson M., Hörnsten C., Nyqvist F., Padyab M., Nygren B., Olofsson B., Lövheim H., Gustafson Y. (2015). Psychometric properties and feasibility of the Swedish version of the Philadelphia Geriatric Center Morale Scale. Qual. Life Res..

[20] Pinar R., Oz H. (2011). Validity and reliability of the Philadelphia Geriatric Center Morale Scale among Turkish elderly people. Qual. Life Res..

[21] Nagata A., Yamagata Z., Nakamura K., Miyamura T., Asaka A. (1999). Sex Differences in Subjective Well-Being and Related Factors in Elderly in the Community Aged 75 and Over. Jpn. J. Geriat..

[22] Fujiwara Y., Suzuki H., Yasunaga M., Sugiyama M., Ijuin M., Sakuma N., Inagaki H., Iwasa H., Ura C., Yatomi N. (2010). Brief screening tool for mild cognitive impairment in older Japanese: Validation of the Japanese version of the Montreal Cognitive Assessment. Geriatr. Gerontol. Int..

[23] Nasreddine Z.S., Phillips N.A., Bédirian V., Charbonneau S., Whitehead V., Collin I., Cummings J.L., Chertkow H. (2005). The Montreal Cognitive Assessment, MoCA: A brief screening tool for mild cognitive impairment. J. Am. Geriatr. Soc..

[24] Zitser J., Allen I.E., Falgàs N., Le M.M., Neylan T.C., Kramer J.H., Walsh C.M. (2022). Pittsburgh Sleep Quality Index (PSQI) responses are modulated by total sleep time and wake after sleep onset in healthy older adults. PLoS ONE.

[25] Horikoshi K., Fujita M., Shimadu N., Takashima K. (2021). Life, mental, and social functional factors associated with a decreased activity in elderly requiring support/care. Nippon Ronen Igakkai Zasshi.

[26] Zhang L., Li Z. (2020). A Mokken scale analysis of the Kessler-6 screening measure among Chinese older population: Findings from a national survey. BMC Geriatr..

[27] Haeuchi Y., Honda T., Chen T., Chen S.M., Kumagai S. (2016). Association between participation in social activity and physical fitness in community-dwelling older Japanese adults. Jpn. J. Public Health.

[28] Wilson R.S., Boyle P.A., Segawa E., Yu L., Begeny C.T., Anagnos S.E., Bennett D.A. (2013). The influence of cognitive decline on well-being in old age. Psychol Aging.

[29] Kirova A.M., Bays R.B., Lagalwar S. (2015). Working memory and executive function decline across normal aging, mild cognitive impairment, and Alzheimer’s disease. BioMed Res. Int..

[30] Cooper C., Bebbington P., Livingston G. (2011). Cognitive impairment and happiness in old people in low and middle income countries: Results from the 10/66 study. J. Affect. Disord..

[31] Yochim B.P., Mueller A.E., Segal D.L. (2013). Late life anxiety is associated with decreased memory and executive functioning in community dwelling older adults. J. Anxiety Disord..

[32] Frankenberg C., Weiner J., Knebel M., Abulimiti A., Toro P., Herold C.J., Schultz T., Schröder J. (2021). Verbal fluency in normal aging and cognitive decline: Results of a longitudinal study. Comput. Speech Lang..

[33] Hashimoto S., Yamashita S., Uno H. (2021). A study on the relationship between communication and the purpose in life of the elderly. J. JSCE.

[34] Bellucci G. (2020). Positive attitudes and negative expectations in lonely individuals. Sci. Rep..

